# Licorice protects against ulcerative colitis via the Nrf2/PINK1‐mediated mitochondrial autophagy

**DOI:** 10.1002/iid3.757

**Published:** 2022-12-28

**Authors:** Jinrong Kong, Qingzhen Xiang, Gaoxiang Shi, Zaiping Xu, Xiaowen Ma, Yunlai Wang, Zihua Xuan, Fan Xu

**Affiliations:** ^1^ School of Pharmacy Anhui University of Chinese Medicine Hefei People's Republic of China; ^2^ Anhui Province Key Laboratory of Chinese Medicinal Formula Hefei People's Republic of China

**Keywords:** licorice, mitochondrial autophagy, Nrf2, PINK1, ulcerative colitis

## Abstract

**Purpose:**

Study of the effects and mechanisms of licorice in the treatment of ulcerative colitis (UC) from the perspective of mitochondrial autophagy.

**Methods:**

BALB/C mice were induced with 3% dextran sodium sulfate to build an animal model of UC. After 7 days of modeling, different doses of licorice were administered for 7 days. Hematoxylin and eosin staining is used to detect pathological changes in the colon. Mitochondrial membrane potentials and reactive oxygen species (ROS) contents were detected by flow cytometry, and autophagy of mitochondria was observed by transmission electron microscopy. Determination of inflammatory cytokines by enzyme‐linked immunosorbent assay. The oxidizing factors are detected by the kits. Western blot analysis was used to detect expressions for nuclear factor called erythropoietin (Nrf2), pten‐induced protein kinase 1 (PINK1), Parkin, HO‐1, P62, and LC3.

**Results:**

Licorice improved the pathological condition of UC mice, increasing the mitochondrial membrane potential and decreasing the ROS content. Promotes the emergence of autophagosomes and autophagosomes. The contents of interleukin (IL)‐1β, IL‐6, IL‐17, and tumor necrosis factor‐alpha were downregulated, the contents of superoxide dismutase and glutathione peroxidase were upregulated and the contents of malondialdehyde were downregulated. In addition, licorice promotes the expression of Nrf2, PINK1, Parkin, HO‐1, P62, and LC3.

**Conclusion:**

Licorice was shown to reduce levels of inflammatory factors and oxidative stress in mice with UC, possibly by promoting mitochondrial autophagy through the activation of the Nrf2/PINK1 pathway.

## Graphical abstract

The role of mitochondrial autophagy in ulcerative colitis nuclear factor called erythropoietin promotes mitochondrial autophagy Chinese medicine for ulcerative colitis Look for drugs with fewer side effects for ulcerative colitis To explore new ways to treat ulcerative colitis.

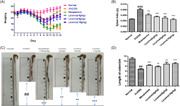

## 1 INTRODUCTION

Ulcerative colitis (UC) is a form of inflammatory bowel disease (IBD) that originates in the rectum and extends from the distal end to the proximal end of the colon, affecting millions of people worldwide and characterized by a tendency to become refractory, relapsing, and cancerous.^1^, ^2^, ^3^ Even though the pathogenesis of UC remains primarily unknown, the combination of environmental factors, genetic factors, mitochondrial dysfunction, intestinal microbiota modifications, and immune responses has been considered involved in the development of UC.^4^, ^5^, ^6^ Most recently, expression of proinflammatory mediators such as reactive oxygen species (ROS), neutrophil infiltration, and cytokines has been known to contribute to the inflammatory cascade in the pathological process of colitis.^7^ The most current standard therapy for UC includes aminosalicylates (e.g., sulphasalazine and mesalamine), glucocorticosteroids (e.g., prednisolone and budesonide), immunosuppressant (e.g., azathioprine, 6‐mercaptopurine, and methotrexate), and biologic agents (e.g., infliximab and adalimumab).^8^ These treatments are effective, however, they might cause severe side effects, including diarrhea, cramps, abdominal pain accompanied by fever, and elevated blood pressure.^9^ Therefore, alternative therapies or drugs with low side effects are needed for the prevention or treatment of UC. Less adverse reactions, the extreme safety of Chinese medicine has been increasingly used in the treatment of diseases.^10^

Licorice (Glycyrrhiza Uralensis Fisch or GanCao) is derived from the dry roots and rhizomes of the leguminous plants Glycyrrhiza Uralensis Fisch, Glycyrrhiza Infatata Bata, or Glycyrrhiza Glabra, and is widely considered as an herb and traditional food in Asia. Currently, licorice remains one of the most commonly prescribed drugs for a variety of conditions such as microbial infections, inflammation, and cancer, and is a plant commonly used in folk and eastern medicine for stomach ulcers, bronchitis, and sore throats. Various polyphenols were isolated from licorice, including phenolic acids, flavonoids, flavonoids, chalcones and isoflavones.^11^ In recent years, a large number of studies have reported that the active components isolated from licorice have anti‐inflammatory, antioxidant, antivirus, antitumor, antibacterial, immunomodulatory, and other activities that restore and protect the nervous, digestive, respiratory, endocrine, and cardiovascular systems. A recent study showed that chemicals in licorice can regulate the oxidative stress system by activating the nuclear factor called erythropoietin 2 (Nrf2), thereby reducing the concentration of ROS in the body. Nrf2 is thought to be a key regulator of inflammatory diseases. This protects different types of cells and tissues from inflammation and oxidative stress, and has been found to be involved in UC pathogenesis. It is, therefore, considered an ideal target for UC therapy. Nrf2 can regulate pten‐induced protein kinase 1 (PINK1) expression under oxidative stress, and PINK1 plays an essential role in the regulation of mitochondrial function. At the same time of ATP output, mitochondria are easily injured by ROS, which leads to a series of pathophysiological processes. Damaged mitochondria also accumulate genetic damage and export harmful sances such as ROS to the cytoplasm.^12^, ^13^ An increase in ROS leads to an increase in the severity of inflammation, while an excess of ROS leads to impaired mitochondrial function and increased inflammation. Under stress conditions, the mitochondrial membrane potential is reduced by mitochondrial dysfunction or by the opening of percolation transition pores. As a result, PINK1 cannot be transferred smoothly to mitochondria, which are attached to the surface of mitochondria.^14^ PINK1, which is activated on the surface of the mitochondria, calls up the cytokine Parkin. Through its E3 ubiquitin ligase activity, Parkin conjugates ubiquitin to proteins on the outer mitochondrial membrane (OMM), which amplifies a signaling cascade culminating in mitochondrial engulfment by a double‐membrane structure called an autophagosome. The fusion of an autophagosome with a lysosome degrades the damaged mitochondrion and permanently removes it from the normal population. This process is known as mitochondrial autophagy. Mitochondrial function can be improved by scavenging ROS and enhancing mitophagy.^15^

Mitochondrial autophagy refers to the selective encapsulation and degradation of damaged mitochondria by autophagy, which plays an essential role in maintaining the stability of mitochondrial mass and quantity.^16^

Nrf2 can upregulate PINK1 expression, and PINK1‐regulated mitochondrial autophagy plays an essential role in the expression of inflammatory properties. The active component of licorice can upregulate the expression of PINK1‐induced mitophagy, thus reducing the inflammatory response.^17^ Therefore, this study aimed to evaluate the therapeutic effect of licorice on dextran sodium sulfate (DSS)‐induced UC and to further explore its possible mechanism.

## 2 MATERIALS AND METHODS

### 2.1 Animals

Sixty healthy 6‐ to 8‐week‐old male mice (20 ± 2 g) were obtained from Jiangsu Huangchuang Sino Pharmaceutical Technology Co., Ltd. (SCXK (Su) 2020‐0009). Mice were kept in a 12 h light/dark cycle with controlled humidity (50%–70%) and temperature (20–24°C). The research was approved by the university's Institutional Animal Ethics Committee, and the experiments were applied according to the ethical principles of experimental animals (Approval No. 2022007).

### 2.2 Reagents

DSS was purchased from Shanghai Yisheng Biotechnology Co., Ltd. Licorice was purchased from Anhui Guanghe Traditional Chinese Medicine Co., Ltd. Mesalazine sustained release granules were obtained from Aidifa Pharmaceutical Co., Ltd. The superoxide dismutase (SOD) kit, malondialdehyde (MDA) kit, and glutathione peroxidase (GSH‐PX) kit were from Jiancheng Biology Co., Ltd. The mitochondrial membrane potential kit, phosphate buffered saline (PBS), and ROS kit was purchased from Suolaibao Technology Co., Ltd. Enzyme‐linked immunosorbent assay (ELISA) kits were purchased from Jianglai Biotechnology Co., Ltd. glyceraldehyde‐3‐phosphate dehydrogenase (GAPDH) rabbit polyclonal antibody, Nrf2 rabbit polyclonal antibody, P62 rabbit polyclonal antibody, LC3 rabbit polyclonal antibody and horseradish peroxidase (HRP)‐labeled goat anti‐rabbit IgG were obtained from Abcam, PINK1 rabbit polyclonal antibody, Parkin rabbit polyclonal antibody, HO‐1 rabbit polyclonal antibody were purchased from Zhengneng Biotechnology Co., Ltd. roswell park memorial institute (RPMI)1640 (SH30027.FS) was from Hyclone Company, tissue dissociation kit (130‐110‐201).

### 2.3 Quality control of licorice

High performance liquid chromatography (HPLC) analysis after filtration of licorice using a 0.22 µm microporous membrane. Agilent WondaSil C18 Superb was used for chromatographic separation. The mobile phase was composed of acetonitrile (A) and 0.1% phosphoric acid water with a linear gradient elution: 0–15 min, 20%–30% A; 15–25 min, 30%–32% A; 25–30 min, 32%–43% A; 30–43 min, 43%–44% A.) Eluate was monitored by a diode array detector at a wavelength of 254 nm (20 µl, 1.0 ml/m, 30°C). HPLC condition reference Shi et al.^18^

### 2.4 Establishment of UC

Sixty BALB/C mice were reared adaptively for 3 days, 10 mice were randomly selected as the normal group, the remaining mice drank 3% DSS solution (precision to take DSS 3 g, with distilled water volume to 100 ml, alternative conditions are the same). After 7 days, the mice were randomly divided into 3% DSS group, mesalazine group (200 mg/kg/day), licorice low, middle and high group (4, 8, 16 g/kg/day), the drug was given orally continuously for 7 days (normal group was given saline solution). UC model reference Shi et al.^18^

After 7 days of 3% DSS for UC modeling, licorice and mesalazine were gavaged for 7 days, and the body weight of the mice was recorded daily. On the 15th day, after the mice had been killed by taking blood under anesthesia, the spleens were removed and weighed for recording. Colons were removed and the length of the colon measured with a ruler.

### 2.5 Assessment of colon damage by hematoxylin and eosin (H&E)

The colonic tissue was held in a 10% formaldehyde solution. For histological analysis, tissue sections were stained with standard techniques of hematoxylin and eosin. The colons were examined in cross sections at ×200 magnification. This step is referenced by Liu et al.^17^

### 2.6 The morphology of mitochondria in colon tissue was observed by transmission electron microscope (TEM)

Colon tissue was removed and fixed with 2.5% glutaraldehyde for 6 h, then poured into PBS buffer and fixed with 1% acetic acid for 2 h. Dehydration and soaking were carried out with related reagents, and the mitochondria were embedded in a 40°C oven for 12 h and then in a 60°C oven for 48 h. The embedded blocks were cut into ultrathin slices (70 nm). Morphological changes in mitochondria were observed by transmission electron microscopy after recording images on a digital camera stained with lead and uranium. This step is referenced by Mao et al.^19^

### 2.7 Assessment of oxidative stress markers in colon tissue

Colon tissues were weighed and homogenized in 9 volumes (w/v) of ice‐cold physiological saline. The homogenized samples were then centrifuged at 1000*g* for 10 min for GSH‐PX, MDA, and SOD measurements. Standard kits from Nanjing Jiancheng Bioengineering Institute were used to check the oxidative stress markers as directed by the manufacturer. This step is referenced by Mohamed et al.^20^

### 2.8 Detection of cytokines in colon tissue

Colon tissue was homogenized with precooled normal saline. The homogenate was centrifuged at 1000 rpm for 10 min and the supernatant was removed and stored at −80°C until further analysis. Tumor necrosis factor α (TNF‐α), interleukin (IL)‐1β, IL‐6, and IL‐17 levels were analyzed by ELISA. This step is referenced by Mohamed et al.^20^

### 2.9 Flow cytometry measured the levels of ROS

The enzyme mixture of the tissue dissociation kit was prepared, and 100 µl of H enzyme, 500 µl of R enzyme, and 25 µl of An enzyme were added to 4.4 ml of RPMI1640 medium. The tissue is cut into tiny pieces 2–4 mm in diameter. Transfer the tissue to a Gentle MACS C (130‐093‐237) centrifuge tube (on ice) containing the enzyme solution. Tighten tube C and hang it upside down on the sleeve of Gentle MACS dissociator (130‐093‐235). Run h_Tumor_01 in the Gentle MACS dissociator. After terminating the program, remove tube C from Gentle MACS dissociator. Use MACS MIX tube rotator (130‐090‐753) to continuously rotate C tube at 37°C for 30 min. Hang tube C upside down on the sleeve of Gentle MACS dissociator and run the h_Tumor_02 program in Gentle MACS dissociator. Then, place tube C on the MACS Mix tube rotator and rotate continuously for 30 min at 37°C. After termination of the program, remove tube C from Gentle MACS dissociator. After removing the supernatant, the cell precipitate was resuspended using RPMI and filtered using a 70 μm filter (431752) membrane placed on a 50 ml centrifuge tube. Rinse the membrane with a 20 ml RPMI1640 medium, collect the filtrate and centrifuge at 300*g* for 7 min to remove as much supernatant as possible. The cells are resuspended with an appropriate volume buffer, which can be used for subsequent sequencing experiments. The cells were resuspended with 1 ml of precooled PBS. To prepare the probe staining working solution, 10 mM dichlorodihydrofluorescein diacetate (DCFH‐DA) probe solution was diluted with serum‐free medium at 1:1000 to obtain 10 µm staining working solution. Preparation of positive control samples of ROS: Part of the cell suspension was taken, 1 µl positive control Rosup was added and diluted to 1 ml with serum‐free medium at the ratio of 1:1000; the samples were incubated at 37°C for 20 min in the dark. Mix upside down every 3–5 min so that the probe is in full contact with the cell. The cell suspension was centrifuged at 1000 rpm for 5 min, then the supernatant was discarded, and the cells were resuspended with probe staining working solution at a cell concentration of 1 million/ml, and incubated at 37°C for 20 min in the dark. Mix upside down every 3 to 5 min so that the probe is in full contact with the cell. The cells were washed three times with serum‐free medium to completely remove DCFH‐DA that did not enter the cells. Flow cytometry (CytoFLEX) was used for on‐machine detection.

### 2.10 Flow cytometry measured the mitochondrial membrane potential (MMP)

The enzyme mixture of the tissue dissociation kit was prepared, and 100 µl of H enzyme, 500 µl of R enzyme, and 25 µl of An enzyme were added to 4.4 ml of RPMI1640 medium. The tissue is cut into tiny pieces 2–4 mm in diameter. Transfer the tissue to a Gentle MACS C (130‐093‐237) centrifuge tube (on ice) containing the enzyme solution. Tighten tube C and hang it upside down on the sleeve of Gentle MACS dissociator (130‐093‐235). Run h_Tumor_01 in the Gentle MACS dissociator. After terminating the program, remove tube C from Gentle MACS dissociator. Use MACS MIX tube rotator (130‐090‐753) to continuously rotate C tube at 37°C for 30 min. Hang tube C upside down on the sleeve of Gentle MACS dissociator and run the h_Tumor_02 program in Gentle MACS dissociator. Then, place tube C on the MACS Mix tube rotator and rotate continuously for 30 min at 37°C. After termination of the program, remove tube C from Gentle MACS dissociator. After removing the supernatant, the cell precipitate was resuspended using RPMI and filtered using a 70 μm filter (431752) membrane placed on a 50 ml centrifuge tube. Rinse the membrane with a 20 ml RPMI1640 medium, collect the filtrate and centrifuge at 300*g* for 7 min to remove as much supernatant as possible. The cells are resuspended with an appropriate volume buffer, which can be used for subsequent sequencing experiments. The cells were resuspended with 1 ml of precooled PBS. To prepare the probe staining working solution, 10 mM DCFH‐DA probe solution was diluted with serum‐free medium at 1:1000 to obtain 10 µm staining working solution. Preparation of positive control samples of ROS: part of the cell suspension was taken, 1 µl positive control Rosup was added and diluted to 1 ml with serum‐free medium at the ratio of 1:1000; the samples were incubated at 37°C for 20 min in the dark. Mix upside down every 3–5 min so that the probe is in full contact with the cell. The cell suspension was centrifuged at 1000 rpm for 5 min, then the supernatant was discarded, and the cells were resuspended with probe staining working solution at a cell concentration of 1 million/ml, and incubated at 37°C for 20 min in the dark. Mix upside down every 3–5 min so that the probe is in full contact with the cell. The cells were washed three times with serum‐free medium to completely remove DCFH‐DA that did not enter the cells. Flow cytometry (CytoFLEX) was performed.

### 2.11 Western blot analysis

The tissue was cut into pieces, radio immunoprecipitation assay lysate (1 ml/100 mg) and PMSF(10 μl/100 mg) were added, lysed on ice for 30 min, centrifuged at 12,000 rpm for 10 min at 4°C, and the supernatant was collected. The proteolytic products were separated by 10% dodecyl sulfate, sodium salt‐Polyacrylamide gel electrophoresis and transferred to the PVDF membrane by electrophoresis. Then, they were incubated with GAPDH, Nrf2, PINK1, Parkin, HO‐1, P62, and LC3 antibodies at 4°C overnight, incubated with the HRP‐labeled goat anti‐rabbit IgG (1:5000) at room temperature at 1.5 h, and washed with TBST solution three times, each time for 10 min. Using American alternative multifunctional imager detection. The images were quantitatively analyzed using ImageJ.

### 2.12 Statistical analysis

All experiment data were expressed as mean ± SEM. GraphPad Prism 5.0 software was used for statistical and graphical analysis. *p* values were analyzed using analysis of variance, and *p* < .05 is considered significant.

## 3 RESULTS

### 3.1 Quality control of licorice

**Figure 1:**
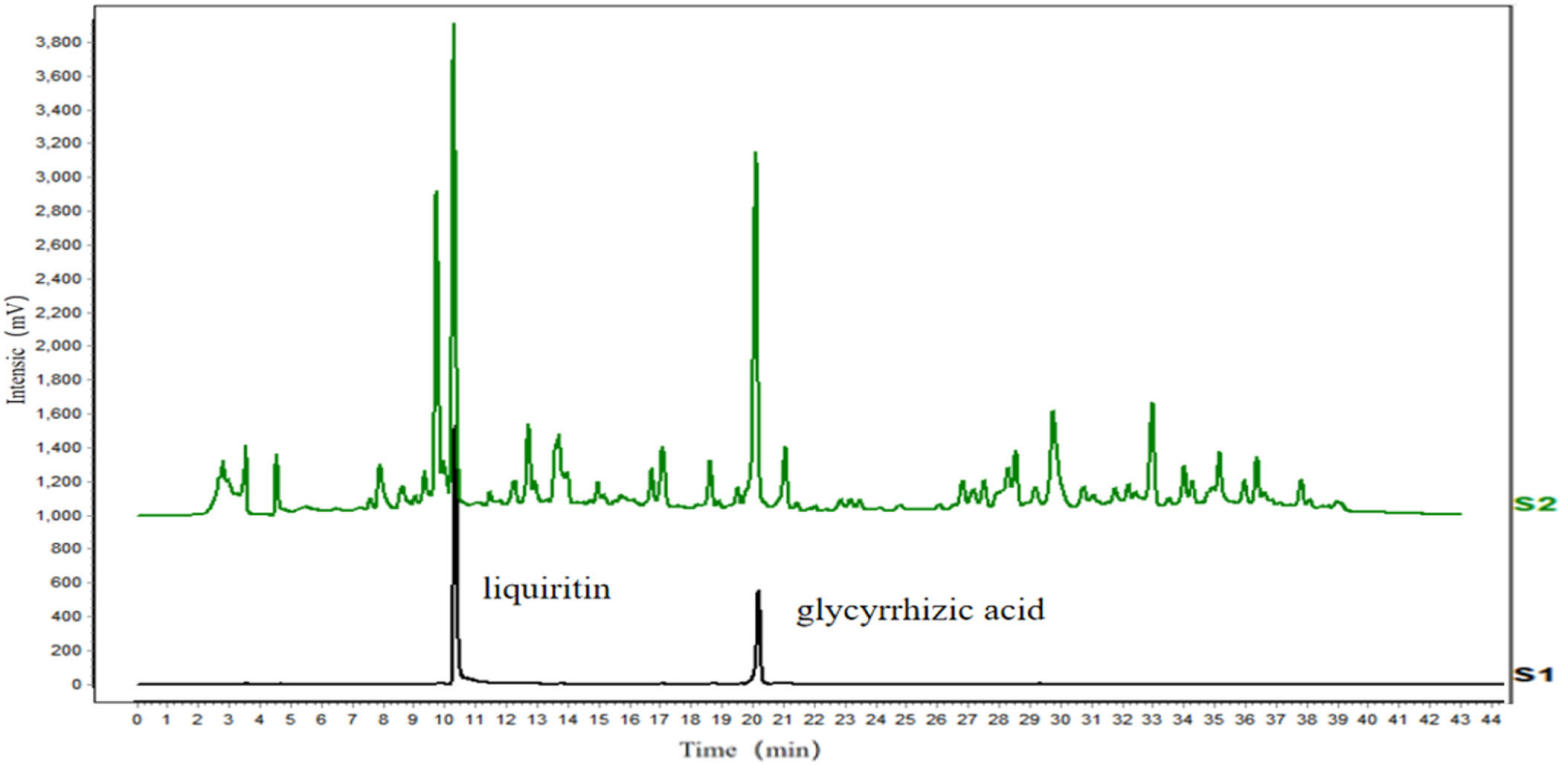
Quality control of licorice. HPLC was used to analyze the main components in the licorice. Sl, mixed standard solution (liquiritin and glycyrrhizic acid); S2, fingerprint of licorice alcohol extract.

The active component of licorice was validated according to the content determination suggested by the Chinese Pharmacopoeia 2020 edition (Chinese Pharmacopoeia Commission, 2020). Two chemical components, liquritin and glycyrrhizic, were chosen as indicators for the ethanol extract of licorice (Figure 1).

### 3.2 Effect of licorice on body weight and colon length

**Figure 2:**
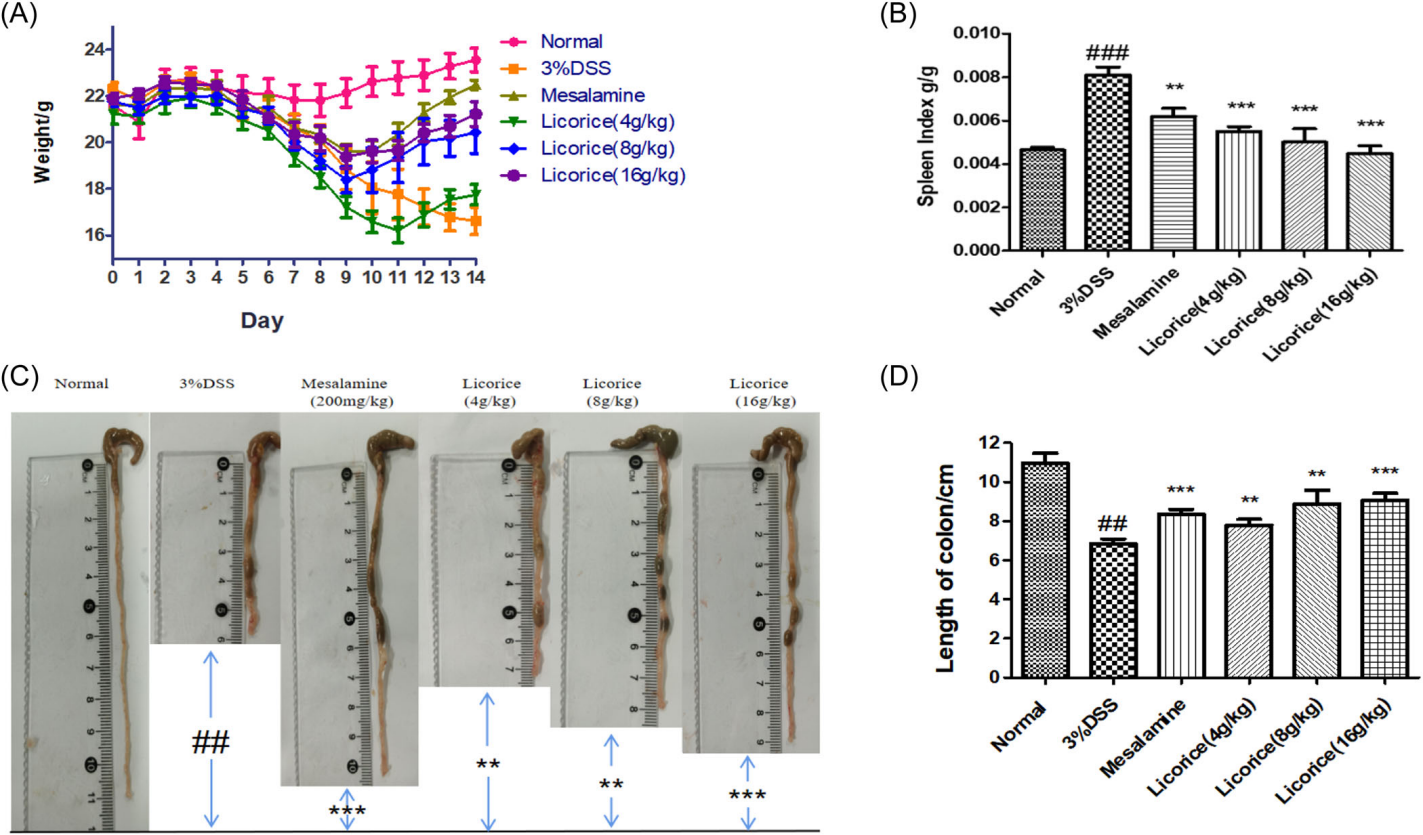
Effects of licorice on body weight, spleen index, and colon length. (A) Weight change; (B) spleen index; (C) photograph of colon; (D) colon length. All values are averaged. These data were analyzed through GraphPad Prism 5. ANOVA followed by Tukey's post hoc test: *n* = 6, ^##^ *p* < .01 versus normal group; **p < .01, and ***p < .001 versus 3% DSS group. ANOVA, analysis of variance; DSS, dextran sodium sulfate.

We observed changes in body weight and colon length in mice. The results showed that the weight of the mice in each group decreased continuously from the 3rd day of modeling. On the 10th day, the mice in the treatment group with 16 g/kg of licorice began to gain weight. On the 10th day, the weight of the mice in the mesalazine group gradually increased, and on the 11th day, the weight of the mice in the treatment group increased by 4 g/kg of licorice. Compared with the 3% DSS group, the spleen index of each group was definitely improved, and the 16 g/kg group was the closest to the normal group (the spleen index is equal to the ratio of the weight of the spleen to the body weight of the day). To investigate whether licorice is involved in the colon shortening induced by 3% DSS, the colon length of mice in the 3% DSS and licorice groups was measured and compared. The licorice group significantly increased the length of the colon, which is inversely proportional to the severity of the disease (Figure 2).

### 3.3 Effect of licorice on histological damnification

**Figure 3:**
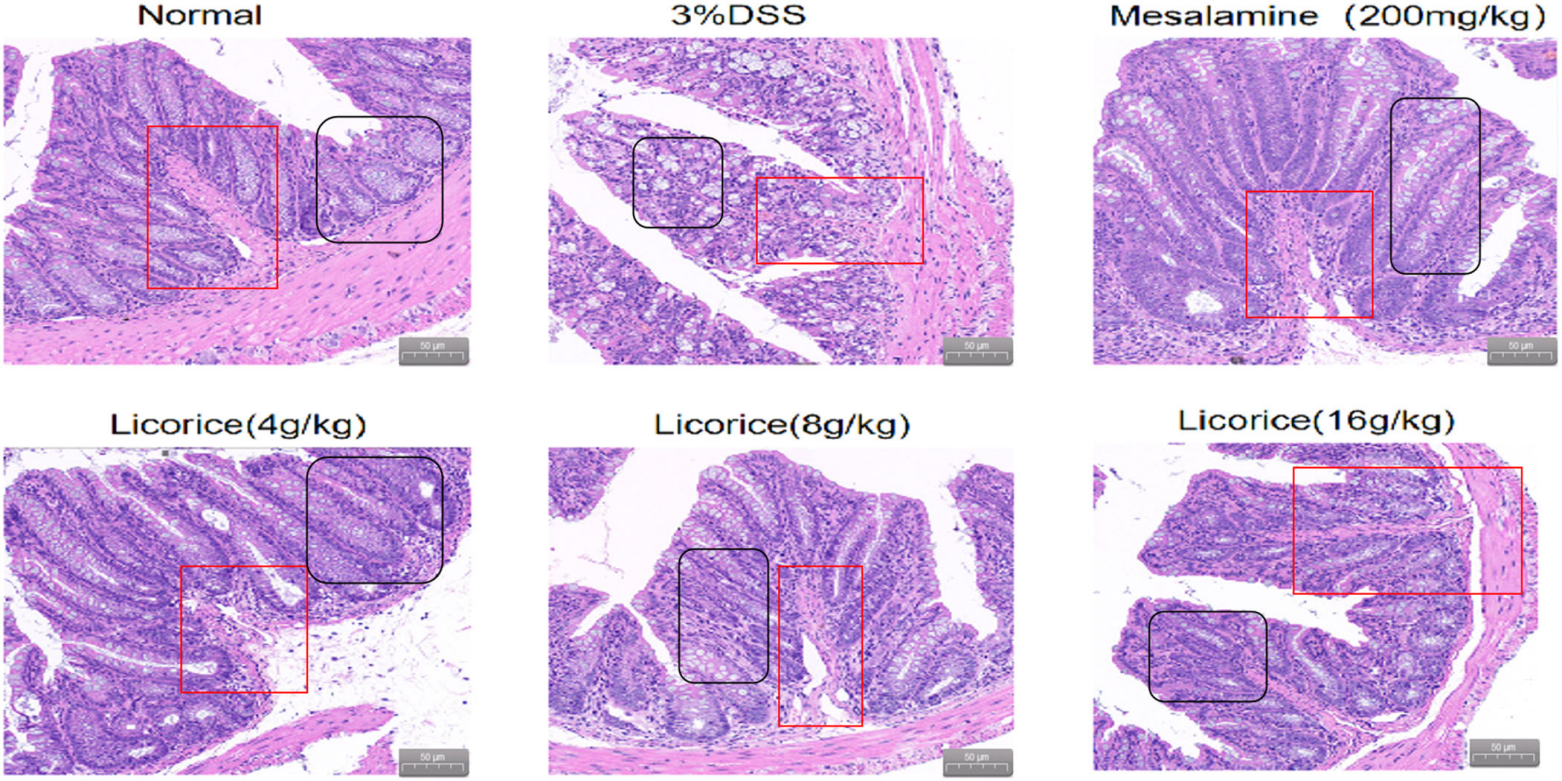
Effect of licorice on histological damnification. Effects of licorice on the histological change in DSS‐induced colitis in mice. The colons were stained with hematoxylin and eosin and the pathological changes were observed (magnification = ×200). The red marks refer to glands and the black marks to inflammatory cells. DSS, dextran sodium sulfate.

The H&E results showed no significant inflammatory infiltration of the colon tissue structure in the normal group. The intestinal mucosa of normal mice was found to be intact, and crypt structures and abundant goblet cells were observed. After 3% DSS, colonic tissue showed marked mucosal ulceration, crypt destruction, and inflammatory cell infiltration. The licorice intervention significantly improved lesions in the colonic tissue. Of these, the 16 g/kg group is the most pronounced (Figure 3).

### 3.4 Effect of licorice on the morphology of mitochondria in colon cells observed by TEM

In the normal population, the colonic epithelial cells are distributed as linear dense lamellae, and the microvilli of the colonic epithelium are arranged in an ordered manner. In 3% of DSS mice, the intestinal barrier was damaged, the intestinal villi were reduced or even lost, and fibrosis appeared. The length, density, and uniformity of the distribution of the villi increases and the gap junction decreases.

**Figure 4:**
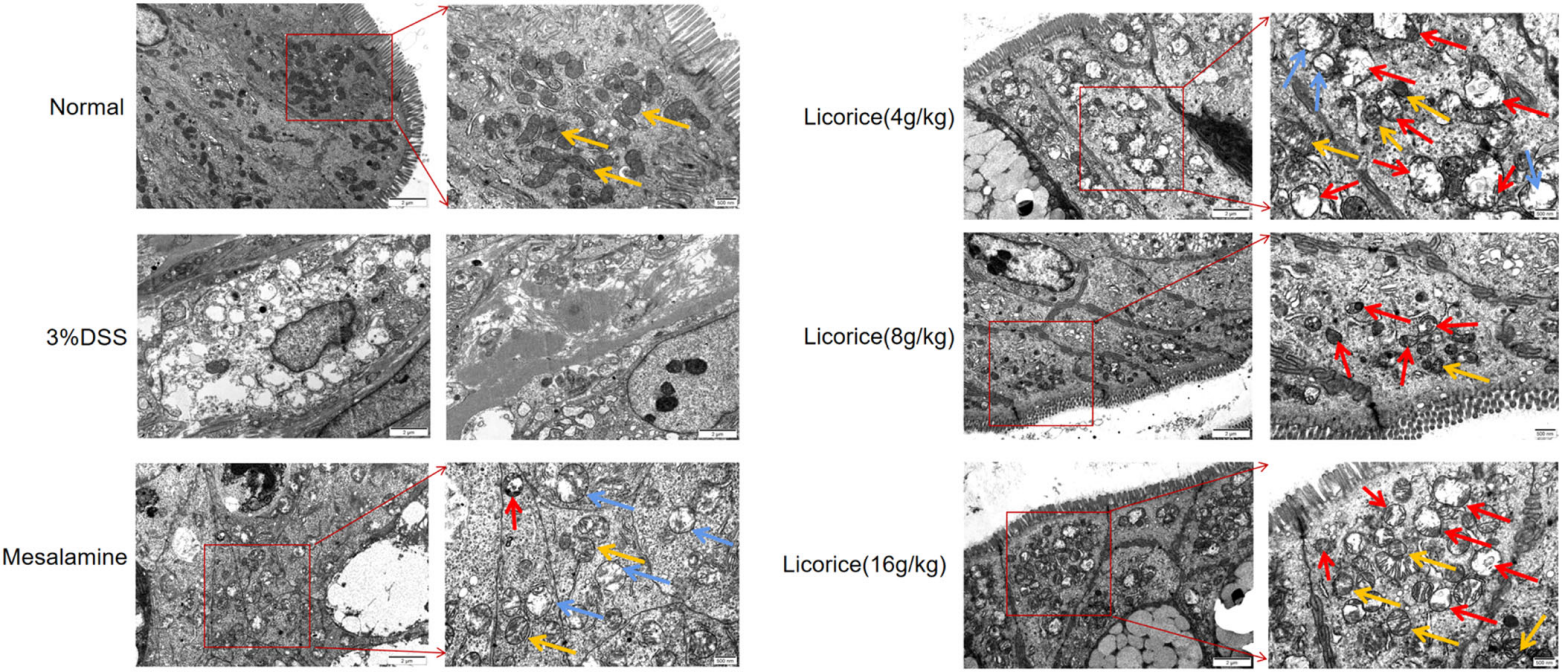
Effect of licorice on the morphology of mitochondria in colon cells observed by TEM. The yellow arrows represent normal mitochondria, the blue arrows represent swollen mitochondria, and the red arrows represent autophagosomes or autophagic lysosomes. The numbers of autophagosomes and autophago lysosomes represent the process of mitochondrial autophagy (magnification = ×25,000, bar = 500 nm). TEM, transmission electron microscope.

The normal group had extra mitochondria and complete mitochondrial cristae in the colonic epithelium. In 3% of DSS mice, the structure of the gut mitochondria was damaged, the number of mitochondria decreased, the degree of mitochondrial swelling increased, the structure of the mitochondria cristae was disordered, and apoptotic corpuscles appeared. After drug treatment, the number of mitochondria increases, the structure of the mitochondria cristae is repaired, the degree of mitochondrial swelling decreases, and autophagy and autophagy lysosomes appear, suggesting the phenomenon of mitochondrial autophagy (Figure 4).

### 3.5 Effect of licorice on oxidative stress markers in colon

**Figure 5:**
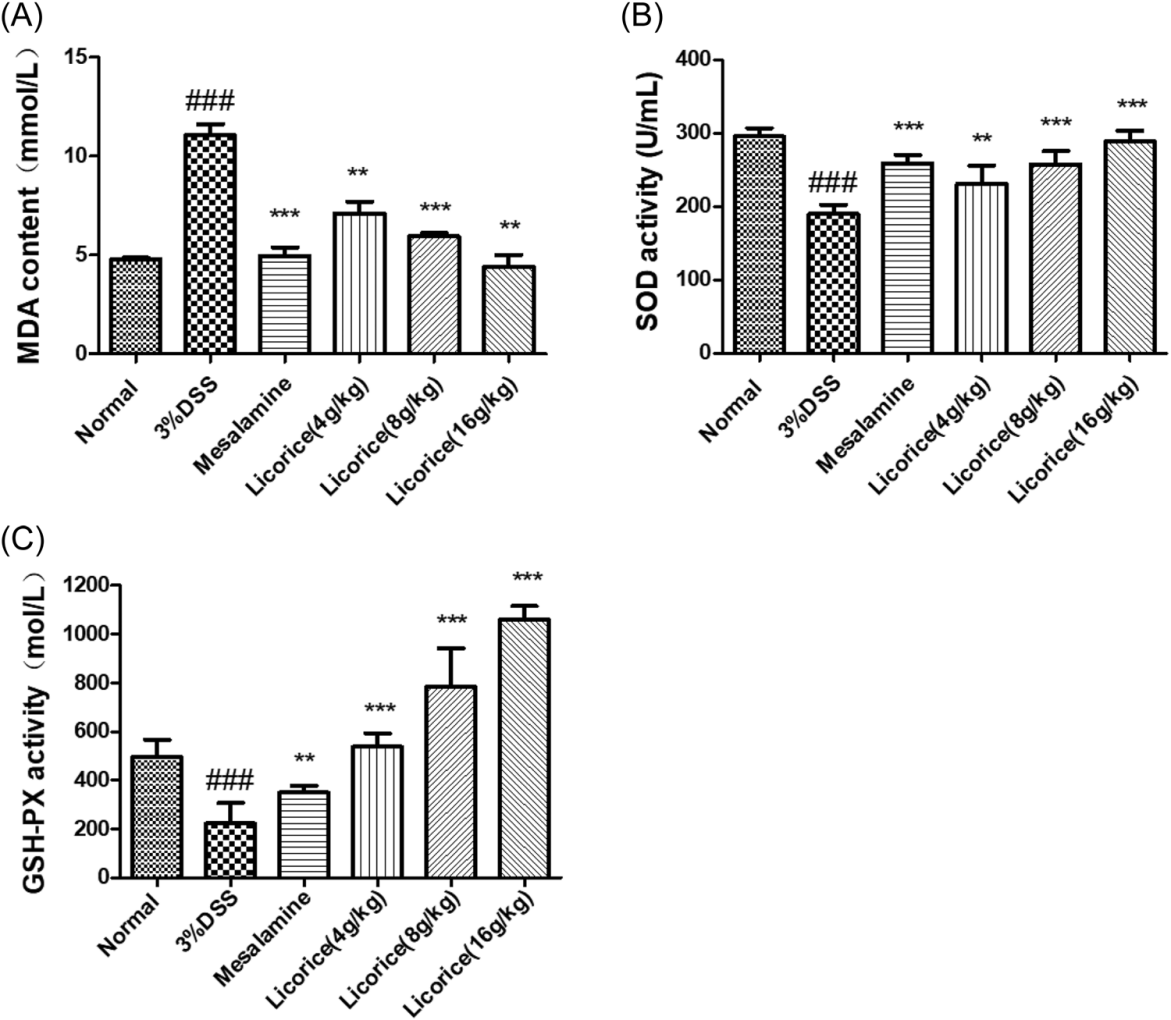
Effect of licorice on the oxidative stress markers in colon. The contents of MDA, SOD, and GSH‐PX in colon tissues were detected by microplate method. (A) MDA; (B) SOD; (C) GSH‐PX. ANOVA followed by Tukey's post hoc test: *n* = 3, ^###^ *p* < .001 versus normal group; *p < .05, **p < .01, and ***p < .001 versus 3% DSS group. ANOVA, analysis of variance; DSS, dextran sodium sulfate; GSH‐PX, glutathione peroxidase; MDA, malondialdehyde; SOD, superoxide dismutase.

As shown in Figure 5, the MDA content increased and the SOD and GSH‐PX content decreased significantly in the 3% DSS group compared to the normal group. Compared to the 3% DSS group, the licorice group had a significantly lower MDA content and a significantly higher SOD content. The MDA content is inversely proportional to the drug concentration and the SOD content is absolutely proportional to the drug concentration. It should be noted that the increase in GSH‐PX in the licorice‐treated mice was extremely significant. The GSH‐PX content of mice in the 4 g/kg group was close to that of the normal group, the content of GSH‐PX in the 16 g/kg group was significantly higher than that in the normal group (Figure 5).

### 3.6 Effect of licorice on ROS content in colon tissue

**Figure 6:**
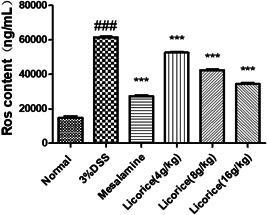
Effect of licorice on ROS in the colon. The levels of ROS in the colon tissue were measured by flow cytometry. ANOVA followed by Tukey's post hoc test: *n* = 3, ^###^ *p* < .001 versus normal group; ***p < .001 versus 3% DSS group. ANOVA, analysis of variance; DSS, dextran sodium sulfate; ROS, reactive oxygen species.

ROS has caused severe damage to the body. To investigate the effect of licorice on ROS in vivo, we examined ROS levels in the colon tissue cells of each group of mice by flow cytometry. As it turned out, ROS levels in the mice were significantly reduced after treatment. There was an inverse relationship between licorice concentration and ROS levels in mice treated with licorice. The results showed that after treatment with licorice, the amount of oxidized material in each group decreased. This suggests that licorice improves oxidative stress in mice (Figure 6).

### 3.7 Effect of licorice on the secretion of cytokines in colon tissues

**Figure 7:**
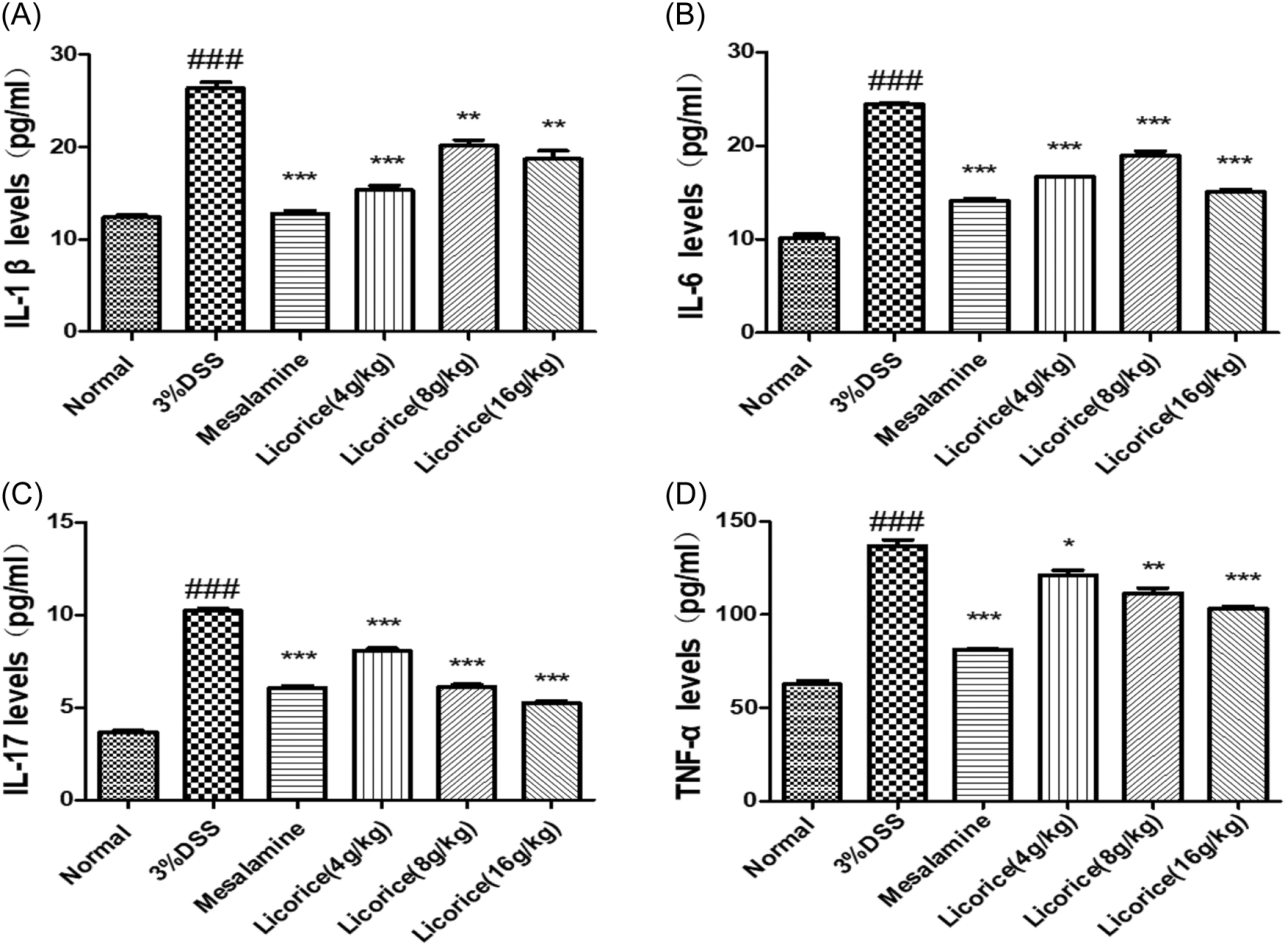
Effect of licorice on the secretion of cytokines in colon. ELISA was performed to measure the secretion levels of IL‐1β, IL‐6, IL‐17, and TNF‐α in the colon tissue. (A) IL‐1β; (B) IL‐6; (C) IL‐17; (D) TNF‐α. ANOVA followed by Tukey's post hoc test: *n* = 3, ^###^ *p* < .001 versus normal group; *p < .05, **p < .01, and ***p < .001 versus 3% DSS group. ANOVA, analysis of variance; DSS,  dextran sodium sulfate; ELISA, enzyme‐linked immunosorbent assay; IL, interleukin.

To investigate the effect of licorice on the release of proinflammatory cytokines from the colon, colon tissue was collected, and ELISA was used to detect the expression of proinflammatory cytokines. In colon tissue, the expression of the cytokines TNF‐α, IL1β, IL‐6, and IL‐17 in the 3% DSS group was significantly higher than that in the normal group. However, the licorice‐treated mice showed a reduction in proinflammatory cytokine expression compared to the 3% DSS group. These results indicated that licorice (16 g/kg) was superior to alternative doses in improving the colon tissue quality and significantly inhibited the 3% DSS‐induced colon proinflammatory cytokine release (Figure 7).

### 3.8 Effect of licorice on MMP in colon tissues

Hundred milligram of colorectal tissue was purified according to the mitochondrial isolation kit procedure for mitochondrial membrane potential (MMP) detection.

**Figure 8:**
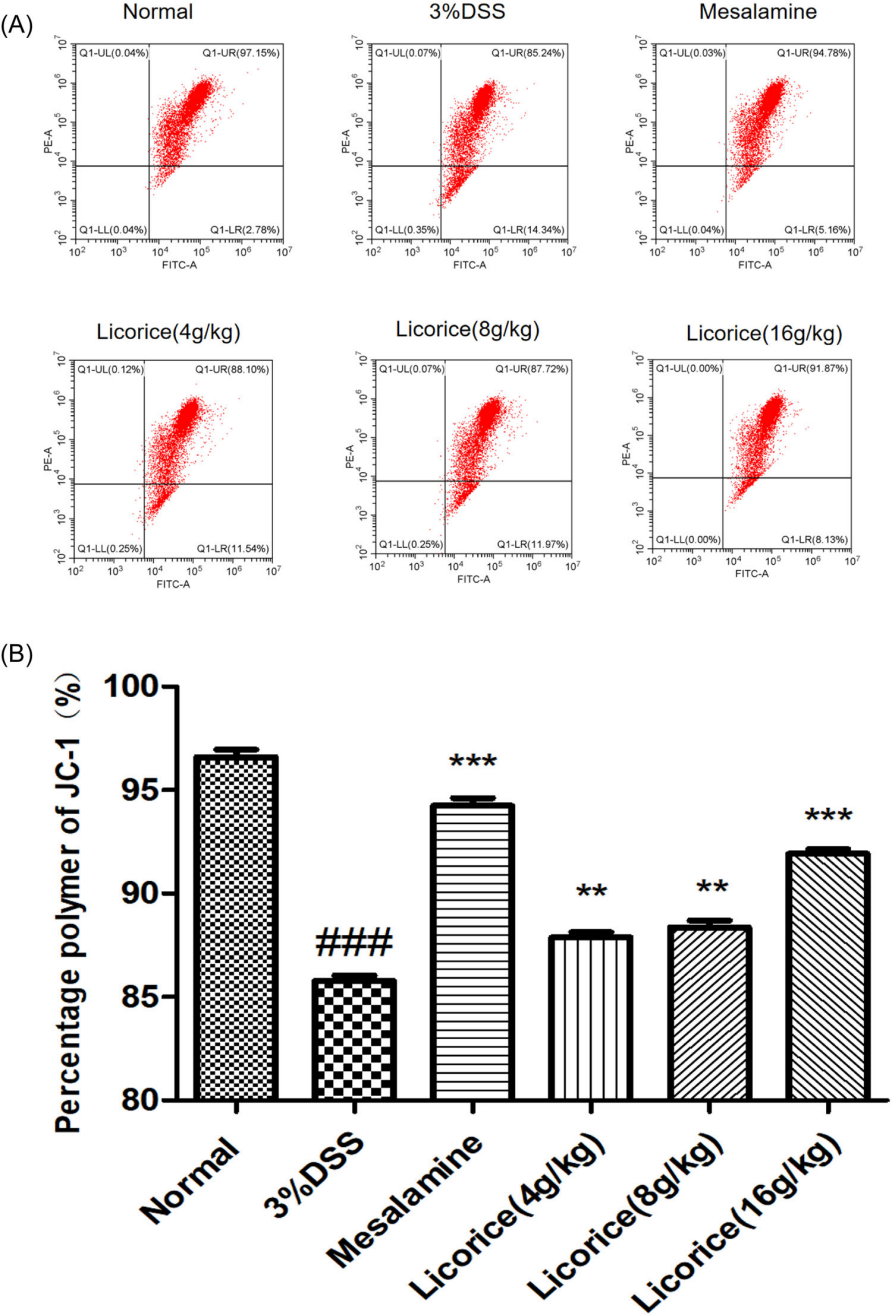
Effect of licorice on MMP in colon. The levels of MMP in the colon tissue were measured by flow cytometry. (A) The flow cytometry of quadrant diagram, (B) Statistical bar chart. ANOVA followed by Tukey's post hoc test: *n* = 3, ^###^ *p* < .001 versus normal group; *p < .05, **p < .01, and ***p < .001 versus 3% DSS group. ANOVA, analysis of variance; DSS, sodium dextran sulfate; MMP, mitochondrial membrane potential.

The results showed a significant increase in MMP levels in the colon tissue of mice in the mesalazine group and the licorice group. The increase was most marked in the magazine group. Compared with the low and middle dose of licorice group (4 and 8 g/kg), the licorice group (16 g/kg) had the best effect on the increase of MMP (Figure 8).

### 3.9 Western blot analysis

**Figure 9:**
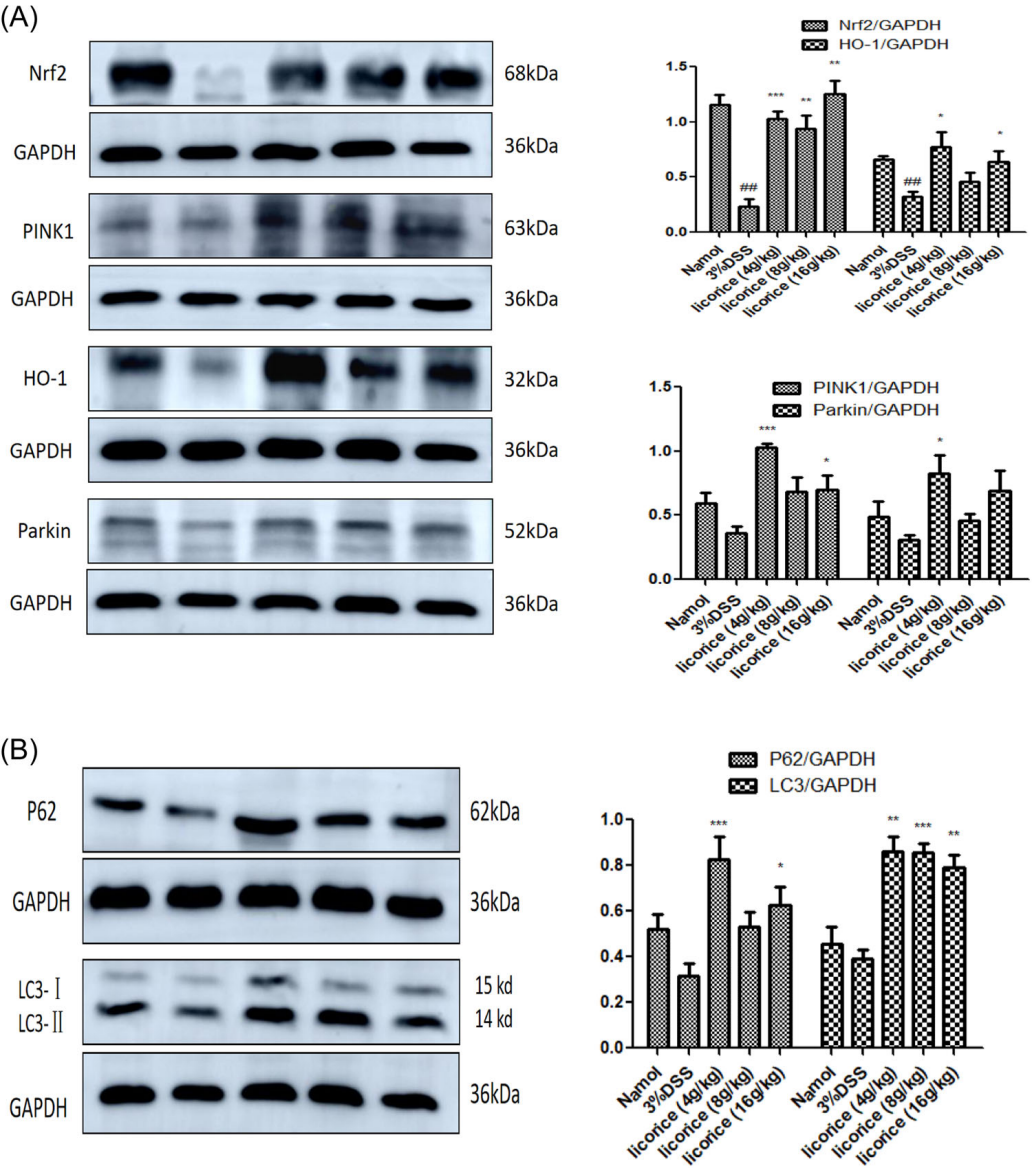
Effect of licorice on the activation of Nrf2/PINK1 pathway in colon. Representative image of Western Blot analysis. (A) Licorice promotes the expression of the Nrf2/PINK1 pathway. (B) Licorice promotes the expression of P62 and LC3. ANOVA followed by Tukey's post hoc test: *n* = 3, ^##^ *p* < .01 versus normal group; *p < .05, **p < .01, and ***p < .001 versus 3% DSS group. ANOVA, analysis of variance; DSS, dextran sodium sulfate; Nrf2, nuclear factor called erythropoietin 2; PINK1, pten‐induced protein kinase 1.

Western blot showed that licorice (4, 8, and 16 g/kg) could upregulate the expression of Nrf2, PINK1, HO‐1, and Parkin proteins in colon tissues, especially in the 4 and 16 g/kg groups (Figure 9A). The results also showed that licorice promotes the expression of P62 and LC3, suggesting that autophagy occurs in colon cells after licorice therapy (Figure 9B).

## 4 DISCUSSION

UC is a slow and extremely recurrent IBD characterized by abdominal pain, diarrhea, mucous pus, and hematochezia. It is very widespread around the world. Inflammatory reactions and disorders of the oxidative stress system are major factors in the development and exacerbation of UC. All known drugs used to treat UC have severe side effects. Therefore, it is essential to develop natural plant drugs with excellent efficacy and few side effects to treat UC.

In this study, we found that licorice did restore weight in DSS‐induced mice, a direct indicator of UC severity. Licorice significantly alleviates colorectal stenosis, improves spleen index, and reduces colorectal injury. We found that in mice with DSS‐induced colitis, the intestinal barrier was severely damaged, and the length of the colon was significantly reduced. Licorice is effective in improving pathological injury. To observe the effect of licorice on inflammatory reaction, we examined the expression of IL‐1β, IL‐6, IL‐17, TNF‐α, and various oxidants. In our study, we found that oral licorice significantly reduced the expression of oxidative factors in mice with DSS‐induced colitis. DSS significantly increased proinflammatory cytokine expression, but oral licorice significantly inhibited it. Thus, licorice had a favorable anti‐inflammatory effect on DSS‐induced colitis in mice. Overinjury of the intestinal antioxidant defense system is associated with the pathogenesis of UC. Pro‐inflammatory cytokine stimulates immune cells to produce reactive oxidative stress, which causes ROS.^21^ Excessive release of ROS and other oxidative substances in the body can disrupt the homeostasis of the oxidative stress system, resulting in disorders of the oxidative stress system,^22^ thereby weakening the immune system and causing serious diseases such as UC.^23^

Nrf2 blocks and mitigates oxidative stress‐induced cell damage and reduces inflammation by modulating the production of anti‐inflammatory cytokines and inducing antioxidant enzymes. It plays an essential role in the protection of intestinal integrity in UC. When exposed to oxidative stress, Nrf2 migrates to the nucleus and protects cells by inducing the expression of multiple antioxidant genes. MDA is a representative product of ROS‐induced lipid peroxidation, which can cause cell metabolism and dysfunction, resulting in cell death.^24^ Therefore, to prevent ROS from causing tissue damage, our bodies have defense mechanisms such as superoxide dismutase, glutathione peroxidase, and other antioxidant enzymes. They are combined to overcome tissue damage caused by ROS.^25^ To investigate the role of licorice in oxidative stress in colitis in mice, we examined the expression of Nrf2 and HO‐1 proteins. The results showed that Nrf2 and HO‐1 protein expression were significantly decreased in DSS‐induced mice, but Nrf2 and HO‐1 protein expression were significantly increased in licorice group mice colitis model induced by DSS, Nrf2 and HO‐1 protein expression was significantly decreased in DSS‐induced mice colitis model, Nrf2 and HO‐1 protein expression was significantly increased in licorice group mice. At the same time, licorice significantly inhibits MDA levels, increases SOD and GSH‐PX levels, and reduces oxidative stress‐induced colorectal injury.

The PINK1‐Parkin pathway is a key pathway that regulates mitochondrial autophagy. Mitochondrial dysfunction in UC animals can be ameliorated by activation of the PINK1‐Parkin pathway, which promotes mitochondrial autophagy. PINK1 recruits Parkin into the outer membrane of damaged mitochondria to form multiple ubiquitin chains, which are then activated and replenished by the damaged mitochondria. When mitochondria are functioning properly, the number of mitochondrial phagosomes that can be absorbed by the outer MMP is limited. PINK1 recruits Parkin to enter the outer membrane of the damaged mitochondria, triggering mitochondrial phagocytosis in the damaged cells, which is equivalent to a self‐protective response.^26^, ^27^ When licorice increased Nrf2, PINK1 increased, and increased PINK1 recruits additional Parkin and promotes mitochondrial autophagy. Therefore, licorice can promote mitochondrial autophagy via the Nrf2‐PINK1 pathway. To further understand the mechanism of regulatory mitochondrial autophagy in licorice in the treatment of DSS‐induced colitis, we analyzed key proteins involved in the process of mitochondrial autophagy. The results showed a significant increase in the expression of Nrf2, PINK1, HO‐1, Parkin, P62, and LC3 and a significant decrease in MMP in mice treated with licorice gavage, thus indirectly demonstrating that licorice promotes mitochondria autophagy. In addition, the number of mitochondria in the colon tissue of licorice‐treated mice increased significantly, and autophagy of mitochondria was observed in the colon tissue of licorice‐treated mice by TME. We, therefore, propose that taking licorice modulates the expression of the Nrf2‐PINK1 pathway, promoting mitochondrial autophagy and ultimately improving DSS‐induced UC.

In previous studies, we investigated the effects of licorice on DSS‐induced colitis in mice by inhibiting the nuclear facter‐kappa B signaling pathway and modulating intestinal microbiota.^18^ Levels of DSS‐induced UC were observed in licorice‐treated mice, validating the ameliorative effects of licorice on UC. There are still some gaps in the research that need to be followed up. For example, different aspects of mitochondrial function have not been further investigated. Additional animal and cell experiments may be needed to further confirm our results. In addition, no effect of measles on the relevant proteins was observed in the WB experiment. This is because in pre‐experiment WB, the authors found that mesalazine did not have a significant effect on the expression of mitochondrial autophagy‐related proteins in the mesalazine group, which may be related to the fact that mesalazine does not treat UC by promoting mitochondrial autophagy. Furthermore, the long‐term effects of licorice intervention on colorectal injury are not known in our current study. Mechanically, our study found that licorice intervention significantly promotes mitochondrial autophagy after colon inflammation by enhancing the Nrf2/PINK1 pathway, suggesting that licorice may be a promising drug for the treatment and management of UC.

## AUTHOR CONTRIBUTIONS

Jinrong Kong is mainly responsible for the content of the experiment and manuscript writing. Gaoxiang Shi, Qingzhen Xiang, Zaiping Xu, and Xiaowen Ma are mainly involved in data analysis research. Fan Xu, Zaiping Xu, and Yunlai Wang oversaw the research and reviewed the final manuscript. All authors have read and approved the final manuscript.

## CONFLICT OF INTEREST

The authors declare no conflict of interest.

## Data Availability

The original data supporting the conclusions of this article can be available from the corresponding authors upon request.
